# The Evolving Field of Regenerative Aesthetics: A Review and Case Series

**DOI:** 10.7759/cureus.87878

**Published:** 2025-07-14

**Authors:** Niamh Corduff, Kate Goldie, Frank Lin, Stephen Lowe, Tuck Wah Siew, Vasanop Vachiramon, Yates Y Chao, Indra Lesthari, Beverly Ong-Amoranto, Ting Song Lim, Ho Sung Choi, Wonkyu Hong, Yui Lam

**Affiliations:** 1 Dermatology, RiverEnd Aesthetics, Newtown, AUS; 2 Dermatology, Clinic 77, London, GBR; 3 Plastic Surgery, Eastern Plastic Surgery, Melbourne, AUS; 4 Dermatology, MUSE Clinic, Sydney, AUS; 5 Dermatology, Radium Aesthetics, Singapore, SGP; 6 Dermatology, Mahidol University, Bangkok, THA; 7 Dermatology, CHAO Institute of Aesthetic Medicine, Taipei, TWN; 8 Dermatology, Sano Clinic Bali, Denpasar, IDN; 9 Dermatology, Dr. Beverly Ong-Amoranto Dermatology Clinics, Makati, PHL; 10 Dermatology, Clique Clinic, Petaling Jaya, MYS; 11 Dermatology, PIENA Aesthetic Medical Clinic, Seoul, KOR; 12 Dermatology, Human Dermatology Clinic, Incheon Metropolitan, KOR; 13 Dermatology, Lam Yui Clinic, Hong Kong, HKG

**Keywords:** asian, biostimulation, calcium hydroxyapatite, collagen, inflammation, regeneration

## Abstract

Dermal fillers such as calcium hydroxyapatite-carboxymethylcellulose (CaHA-CMC), polycaprolactone (PCL), and poly-l-lactic acid (PLLA) are increasingly used as ‘biostimulators’ to stimulate native collagen production for longer-lasting aesthetic improvement. Volume replacement should, ideally, renew local tissue architectures and functions, but the replaced volume may not align structurally or functionally with the original tissue. The ability to achieve this regenerative, biostimulatory aesthetic rejuvenation requires a thorough understanding of the principles and mechanisms of tissue regeneration and its proper application. We reviewed the concepts of regenerative medicine, regenerative aesthetics, and biostimulation in the context of PLLA and CaHA and discussed the effects on immunological pathways and neocollagenesis when these materials are used as biostimulators in clinical aesthetics. Additionally, to understand how the concept of regenerative aesthetics is applied in the real world, we present cases demonstrating best practices and outcomes when using CaHA-CMC in a group of 11 Asian patients. Asian physicians’ practices with CaHA-CMC have evolved beyond its volumizing and contouring benefits to its ability to induce regeneration in aging tissues. This has been achieved through the use of CaHA-CMC as monotherapy or in combination with other modalities. Moreover, CaHA-CMC allows physicians to offer a single, minimally invasive product to patients seeking treatment for skin laxity, wrinkles, crepiness, and volume loss while achieving multiple visible aesthetic improvements. Unlike conventional dermal fillers, the ability to leverage the regenerative qualities of CaHA-CMC effectively resolves age-related aesthetic issues in a durable manner using their body's own systems, allowing patients to emphasize their own unique features.

## Introduction

Aesthetic interventions aim to address many aging-related concerns, including smoothing, hydrating, brightening, or evening out skin tone, and firming of aging skin. Dermal fillers are used to replace volume loss in both superficial and deeper layers [1]. Recently, there has been an increase in aesthetic procedures that stimulate the patient’s own collagen production to give a long-lasting replacement for volume loss. Such ‘biostimulators’ include calcium hydroxyapatite carboxymethylcellulose (CaHA-CMC), polycaprolactone (PCL), and poly-l-lactic acid (PLLA). However, the replaced volume may not align structurally or functionally with the original tissue. Ideal volume replacement should be capable of renewing the local tissue in both structure and function to create a healthier tissue architecture with prolonged aesthetic improvements. A thorough understanding of tissue regeneration mechanisms and the application of these principles to aesthetic medicine can help physicians achieve regenerative biostimulatory aesthetic rejuvenation and may ultimately bring about a paradigm shift in how aesthetic patients are treated. In this report, we primarily sought to explore the use of regenerative aesthetics in medical aesthetics, particularly the use of dermal fillers to biostimulate neocollagenesis for skin rejuvenation and address age-related aesthetic concerns. Our secondary aim was to frame this academic concept practically by demonstrating the outcomes of rejuvenating dermal filler treatments in Asian patients.

Regenerative medicine (RM), regenerative aesthetics (RA), and biostimulation are distinct but related concepts. The repair of damaged tissues can take place via a regenerative process that restores lost tissue with normal structure and function or via a replacement process that replenishes lost tissue without trying to restore normal architecture or function [2,3]. Biostimulation was previously referred to as the physiological process of repair that replaces tissue loss and encompasses both regenerative and replacement mechanisms. Ideal regeneration occurs in human fetal wound healing but is lost in the neonate. RM leverages the body’s innate physiological processes to restore a tissue’s normal structure and function, previously damaged or lost through disease, trauma, and/or aging [2,4]. RA recaptures the youthful structure and function of tissues that have lost integrity due to the aging processes, with the endpoint being improved visual outcomes. These outcomes have positive impacts as patients look younger [4]. The biostimulatory products currently used in aesthetic medicine, most commonly CaHA, PLLA, and PCL, induce different biological behaviors and different physiological outcomes. PLLA [5] and PCL [6] work predominantly through pathways that replace lost tissue with elements of the extracellular matrix (ECM), including the main component, collagen. Collagenesis can generate a different structure from that of normal tissues due to other vital ECM components not being replenished in the correct ratios and architectural relationships [7-9]. In contrast, CaHA works via regenerative processes, repairing the original tissues with the various constituents required to recapture the original tissue architecture, and consequently, to restore tissue functionality [2,4,10]. Unfortunately, confusion exists regarding the term ‘biostimulation,’ which encompasses both ‘collagenesis’ (a replacement repair pathway) and ‘regeneration’ (restoration of normal tissue ecosystems). A replacement/repair pathway that leads to predominantly an increase in collagen is the endpoint of inflammatory foreign body responses, whereas the mechanisms underlying regeneration do result in neocollagenesis but can also follow different non-inflammatory pathways.

Aging increases ECM breakdown and skin laxity [11,12], and produces a pro-inflammatory phenotype [13-18]. The skin tissues have disarrayed collagen [19,20], less elastin [21], less blood supply [22], fewer stem cells [4], a thinner dermis, collapsing 3D structures, and aging microenvironments [12]. In aging, fibroblasts that no longer attach to the ECM [19,23-28] undergo quiescence and senescence [29] and produce biochemical cues that sustain collagen degradation [27,30,31]. Tissue replacement with excessive amounts of collagen results in a stiff, mostly dysfunctional [32] ECM that will not support its associated cells. Regeneration restores the ecosystem of a healthy, optimal, and functional ECM that is interdependent on the cellular population it supports. ECM elasticity and flexibility [33-35] provide the dynamic mechanical cues that stem cells and fibroblasts respond to. RA of aging skin works to renew the dynamic architecture of the normal ECM and provide an optimal microenvironment in which the associated cells can thrive. Three pillars of RA are generally described in the literature: scaffolds, bio-cues, and cells [2,4]. By addressing these pillars individually or simultaneously, the ECM can be renewed to produce a pliable, thick, uniformly pigmented skin with improved circulation and a strong barrier function [4]. CaHA-CMC is a regenerative scaffold that stimulates the production of multiple components of the ECM, resulting in increased vascularity, skin pliability, and thickness [2,4,36-39].

Careful product selection is key to achieving optimal aesthetic outcomes. The regenerative properties of CaHA-CMC [4,38] have been clinically leveraged to treat volume loss, augment tissues, and improve skin quality [40-47]. Volume deficits appear immediately corrected by the carboxymethylcellulose (CMC) gel when undiluted or minimally diluted CaHA-CMC is injected, while contact between CaHA microspheres and fibroblasts leads to collagen, proteoglycan, elastin, and angiogenesis over the three months of gel resorption [48], a regenerative effect that may persist for 12-18 months [42,48]. When diluted or hyper-diluted, CaHA-CMC possesses no direct filling properties. Therefore, the aesthetic correction that can persist for up to two years is the result of the regenerative response resulting from treatment. To understand how CaHA-CMC is used to apply the principles of RA in Asian patients in the Asia Pacific (APAC) region, patient cases from private clinical practices were presented, and best practices for CaHA-CMC were discussed.

## Materials and methods

This study was a survey at an advisory board meeting at a hotel in Vietnam. Dermatologists and plastic surgeons from APAC (n=11) and Europe (n=1), who were experienced with treating Asian patients and using CaHA-CMC, attended an informal meeting to discuss cases of Asian patients treated with CaHA-CMC for skin rejuvenation and/or for visible aging manifestations such as volume loss, deficient structural support, uneven contours, wrinkling, and skin roughness. Physicians retrospectively selected photographs of patients who had undergone successful treatments in their routine clinical practice, and only those with visible improvements in skin quality and aging were included in the discussions. Asian patients tend to have aesthetic concerns relating to their skin quality, including skin crepiness, skin wrinkles and fine lines, dyschromias, and acne scarring in the face and neck. CaHA-CMC has been used to resolve or improve these skin quality concerns [48-50]. Patients included in these discussions were followed up for 12 months (some patients may have been photographed only at three, four, and/or six months) and provided their informed consent for the publication of their photographs and treatment details. Ethics approvals were not required as the data were collected retrospectively and were part of routine clinical patient care.

## Results

Aesthetic considerations and practices observed in our physicians’ work

Dose, Dilution, and Delivery

**Figure 1:**
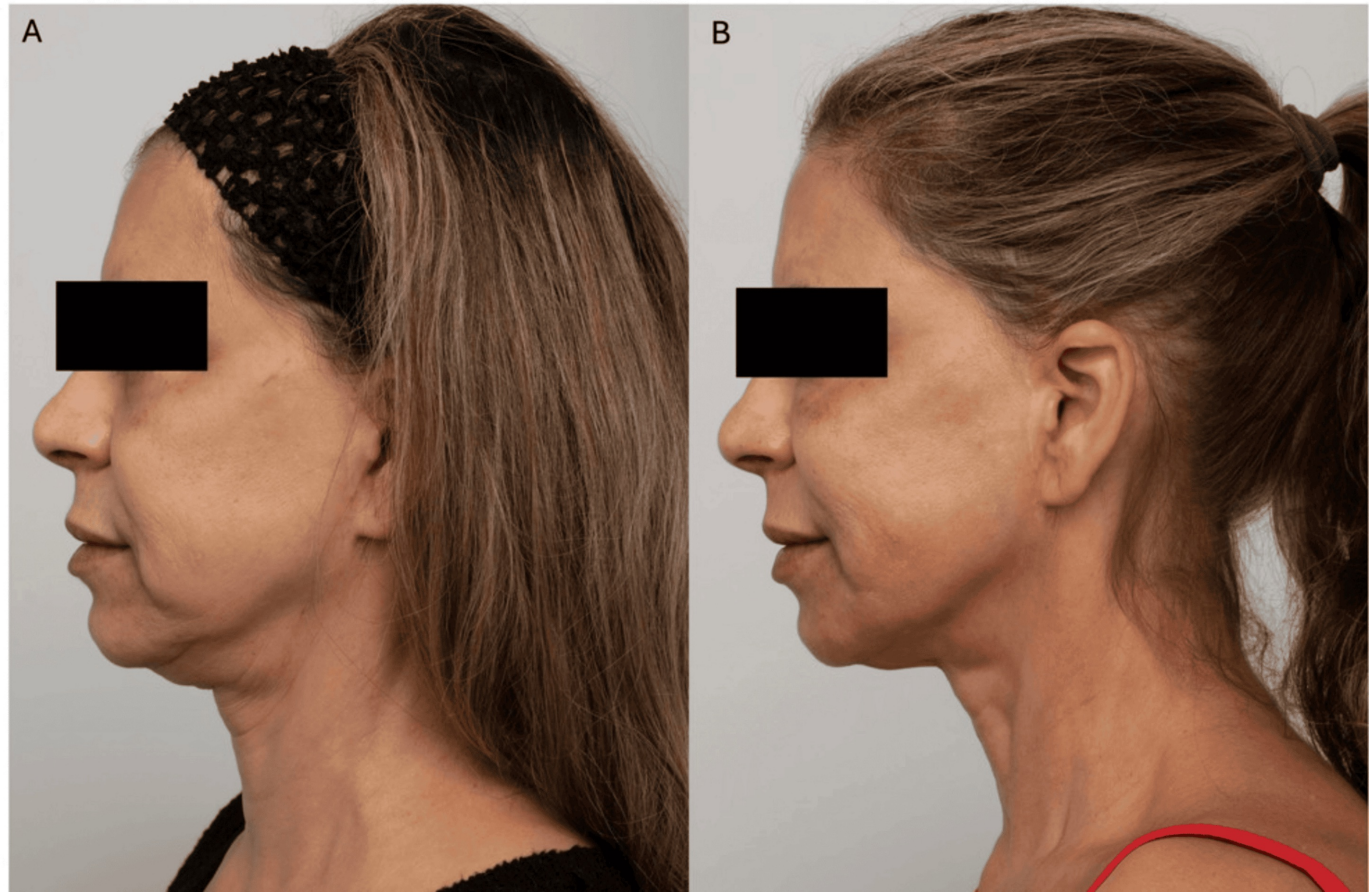
Patient case photographs courtesy of Dr. Stephen Lowe. A 61-year-old female with neck skin quality concerns due to submental laxity, crepey central anterior neck skin, and horizontal lines is shown before (A) and 12 months after (B) neck treatments. One CaHA-CMC (Radiesse® (Merz North America, Raleigh, NC, USA)) syringe was diluted 1:1 per lower face and delivered by subdermal scraping for biostimulation, or diluted 1:2 to increase CaHA particle diffusion, showing long-lasting, global rejuvenation. Written informed consent to include this image in an open-access article was obtained from the patient. CaHA-CMC: calcium hydroxyapatite-carboxymethylcellulose

**Figure 2:**
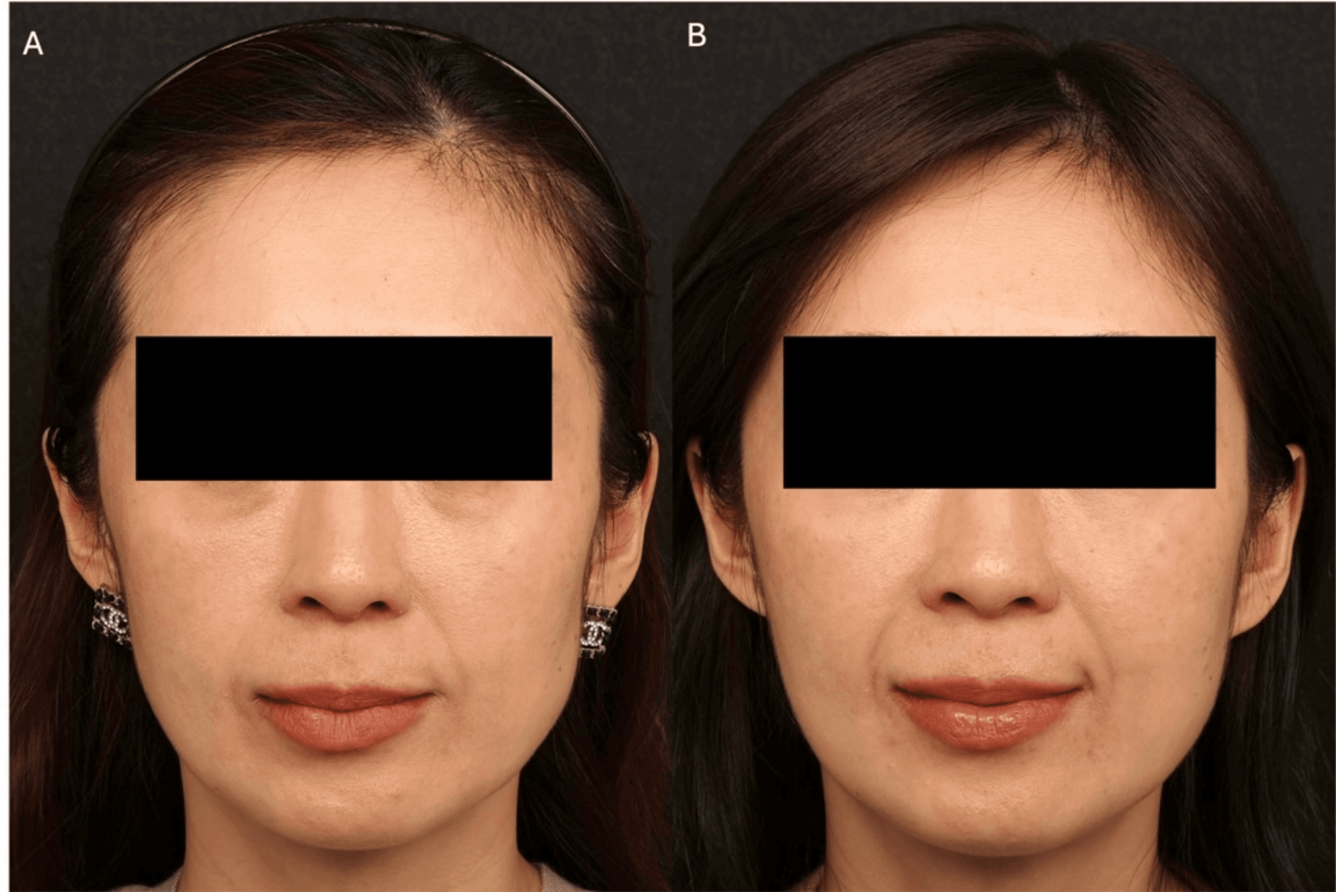
Patient case photographs courtesy of Dr. Frank Lin. Patient shown before (A) and 12 months after (B) treatment. A 42-year-old East Asian female who was very thin, had midface hollowing and significant A-frame deformity and infraorbital hollowing, sought treatment for a tired appearance around the midface and periorbital area. Over two sessions, she received incobotulinumtoxin A (Merz North America, Raleigh, NC, USA) for the masseter and diluted CaHA-CMC for temple contouring and submalar depressions, as well as a CaHA-CMC diluted 1:2 as a mid-cheek pan-facial wash. Undiluted or minimally diluted CaHA-CMC was also placed in the chin and deep pyriform fossa, while hyaluronic acid (Merz North America, Raleigh, NC, USA) was applied to the A-frame and tear trough area. Written informed consent to include this image in an open-access article was obtained from the patient. CaHA-CMC: calcium hydroxyapatite-carboxymethylcellulose

The dose, dilution, and delivery of CaHA-CMC depended on the treated area and targeted tissue depth and whether combination modalities were used. CaHA-CMC is usually diluted 1:1 or 1:2 (i.e., hyperdiluted) and placed in the immediate subdermal plane to boost ECM regeneration [51]. Diluted and hyper-diluted CaHA-CMC increase the diffusion of CaHA particles and were thus used as a biostimulatory “wash” in the neck and submentum (Figure 1) or distributed pan-facially in the mid-cheek and lateral face (Figure 2).

**Figure 3:**
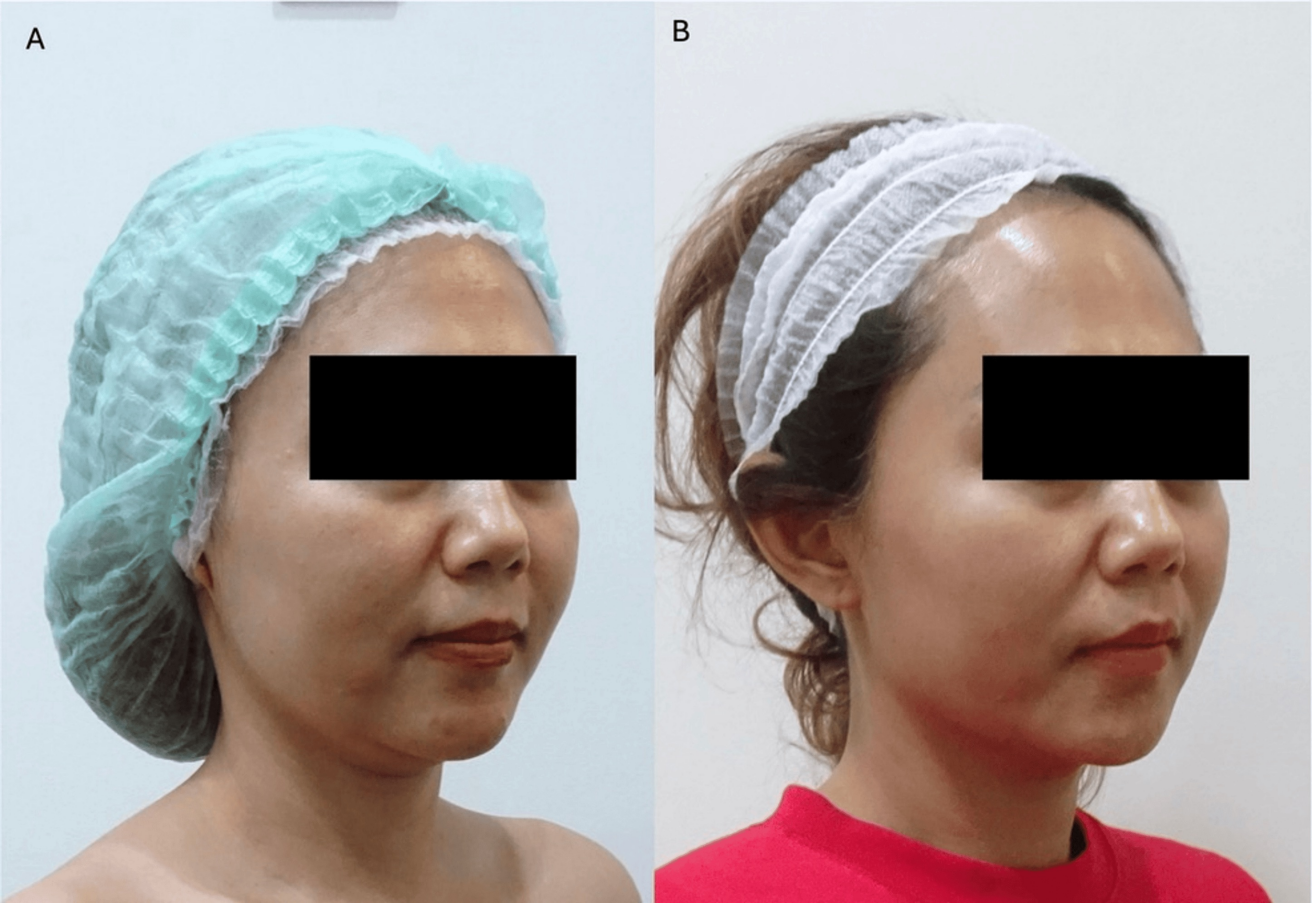
Patient case photographs courtesy of Dr. Indra Lesthari. Patient shown before (A) and at one-year follow-up (B). The patient was a 42-year-old female who requested fillers for aging skin and to improve the midface, lower face, and submental sagging. For cheek augmentation or contouring, undiluted CaHA-CMC with lidocaine was injected deep under the muscle and over the bone in the mid-zygoma (three boluses of 0.05 mL each, in three lines). For the nasolabial folds, two boluses of 0.05 mL each were placed in the subcutaneous layer. One bolus (0.1 mL) was placed deep beneath the mid-cheek muscle, with 0.01-0.05 mL placed slightly subcutaneously for contouring and to pull the skin medially. The remaining undiluted CaHA with lidocaine (up to 0.05 mL) was spread in a thin subcutaneous layer up to the preauricular layer. One syringe of CaHA-CMC was diluted 1:1 and spread to other facial areas, including the upper eyebrow and temple, to tighten the skin and subcutaneous fat, but not to volumize the area, while one syringe of CaHA-CMC (diluted 1:2) was used for submental and neck biostimulation. Written informed consent to include this image in an open-access article was obtained from the patient. CaHA-CMC: calcium hydroxyapatite-carboxymethylcellulose

Undiluted CaHA-CMC can also be used to provide structural support and correct contour deficiencies, thus helping to reverse the visible aging in areas of the mid-face and lower face where volume loss and/or bone resorption was evident, in addition to improving tissue quality and tightening skin with diluted or hyperdiluted CaHA-CMC (Figure 3). Diluted and hyper-diluted CaHA-CMC was delivered using blunt, 22-23G cannulas.

While all physicians reported that the treated indications in their patients were improved or very much improved at three months post-treatment, further improvements manifested as a rejuvenated whole-face or global (including the neck area) appearance at three to six months. By six months, submental laxity, neck horizontal lines, skin crepiness, wrinkles, and texture had all also improved. Together, these aspects contributed to improvements in key skin quality emergent perceptual categories (EPCs) of skin firmness, surface evenness, and radiance [52], all of which persisted beyond six months. Although the Asian consensus recommended treating Asian skin with CaHA-CMC diluted 1:1 [53], results were still very good with hyperdiluted CaHA-CMC (1:2; Figure 4), showing that even at higher dilutions, CaHA-CMC produced positive outcomes [51]. Our group of physicians agreed that the clinical effect of skin tightening seen with diluted CaHA-CMC is partly attributable to its mode of action in strengthening the fibrous septae in subcutaneous tissues, which, in turn, 'repositions' the treated tissues. This is demonstrated in the global pan-facial improvements observed, including skin tightening being directed upwards at the temples and lateral face, as well as submental skin tightening. Our physicians found that among their patients with previous micro-focused ultrasound with visualization (MFU-V) treatment, CaHA-CMC biostimulation improved tired and heavy appearances and seemed to ‘reset’ the skin microenvironment by further stimulating the regenerative process initially induced by energy-based devices. Hyperdiluted CaHA-CMC worked as a potent regenerative biostimulator without adding significant volume and improved skin quality in both the perioral and neck areas (Figure 5), even at one month, with more pronounced improvements at three months.

**Figure 4:**
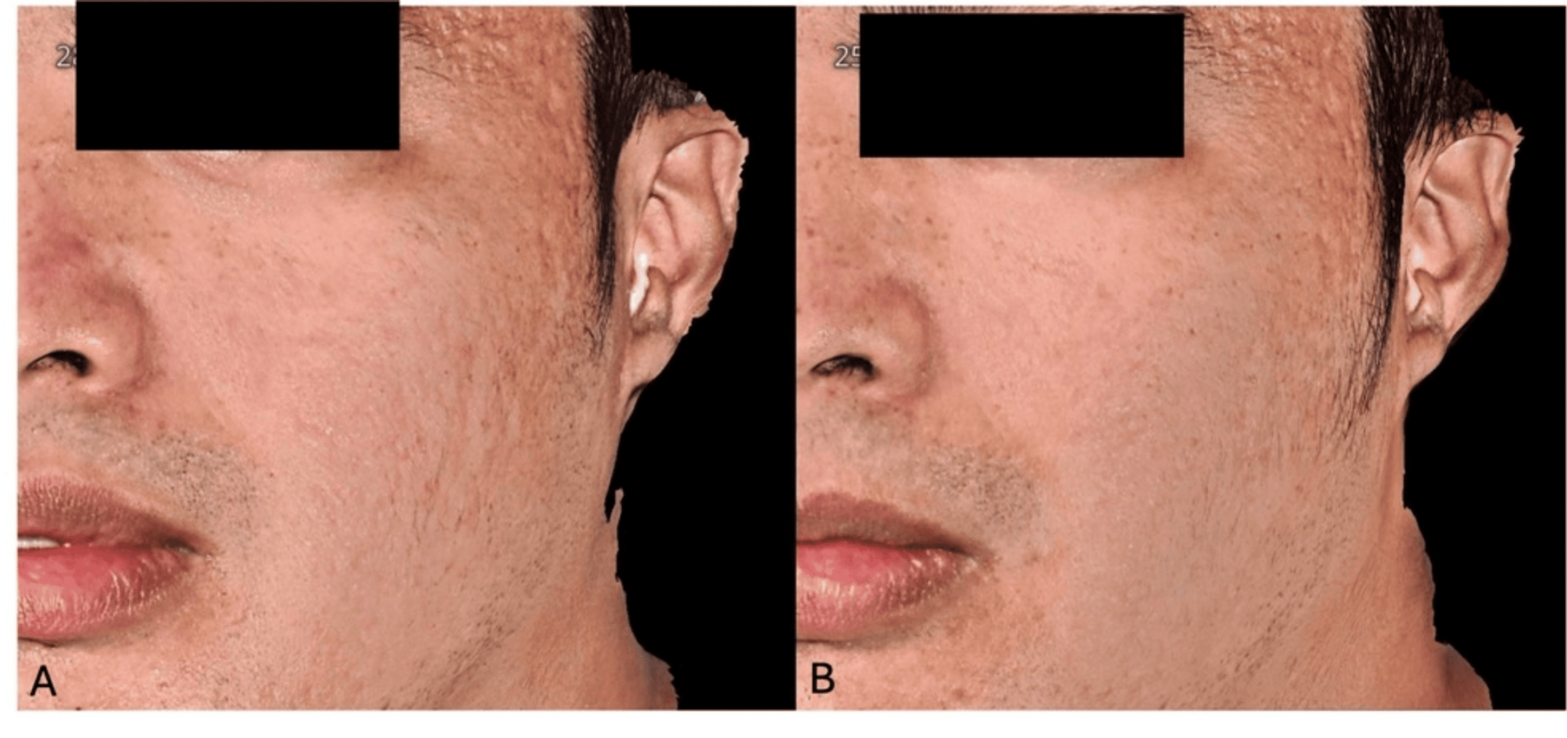
Patient case photographs courtesy of Dr. Beverly Ong Amoranto. A 30-year-old male patient was treated for rolling and boxcar acne scars, shown before (A) and at four months (B) post-treatment with one syringe of hyperdiluted CaHA-CMC (1:2 dilution). Written informed consent to include this image in an open-access article was obtained from the patient. CaHA-CMC: calcium hydroxyapatite-carboxymethylcellulose

**Figure 5:**
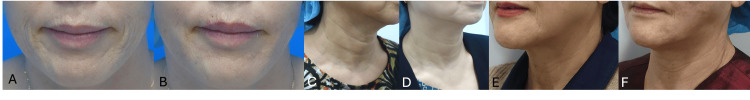
Three patients' photographs courtesy of Dr. Ho Sung Choi. All patients were shown before and three months after treatment. A, B: A 62-year-old female received a 1:1 dilution of CaHA-CMC in the perioral area, followed by undiluted CaHA-CMC (0.3-0.5 mL) for perioral accordion lines. Fine lines on the perioral surface were treated with hyaluronic acid. C, D: A 64-year-old female with neck skin laxity and necklines received two syringes of CaHA-CMC (1:2 dilution) injected slowly and evenly via four to five neck entry points per neck side. E, F: A 72-year-old female patient is shown before and three months after neck rejuvenation. CaHA-CMC (1:2 dilution) was delivered through four to five entry points per neck side through a 23G cannula traveling in the superficial (subdermal) plane and placed in boluses up to 0.02 cc for even distribution. CaHA-CMC: calcium hydroxyapatite-carboxymethylcellulose

Combination Treatments Facilitate Synergistic Effects

**Figure 6:**
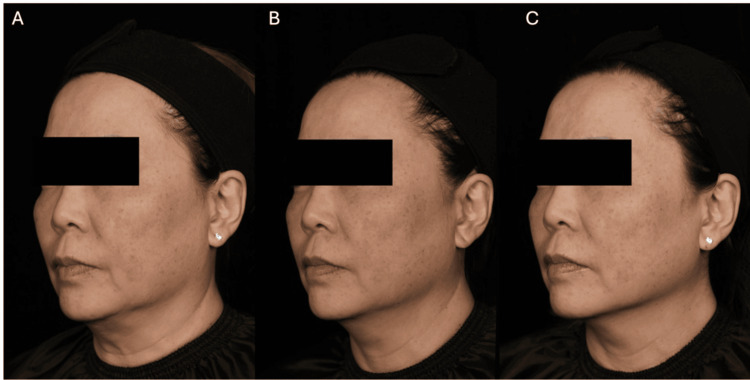
Patient case photographs courtesy of Dr. Tuck Wah Siew. A 58-year-old female with ptosis (jowls), skin laxity and textural abnormalities, facial volume loss, and dyschromia before (A) treatment and three months after receiving a total of 775 MFU-V lines delivered with a 1.5 mm transducer (B), showing visible lifting. Diluted CaHA-CMC (one syringe) was also injected at three months. At a further three months post-CaHA-CMC (C), additional CaHA-CMC and MFU-V-associated lifting and improvements were visible. Written informed consent to include this image in an open-access article was obtained from the patient. CaHA-CMC: calcium hydroxyapatite-carboxymethylcellulose; MFU-V: microfocused ultrasound with visualisation

**Figure 7:**
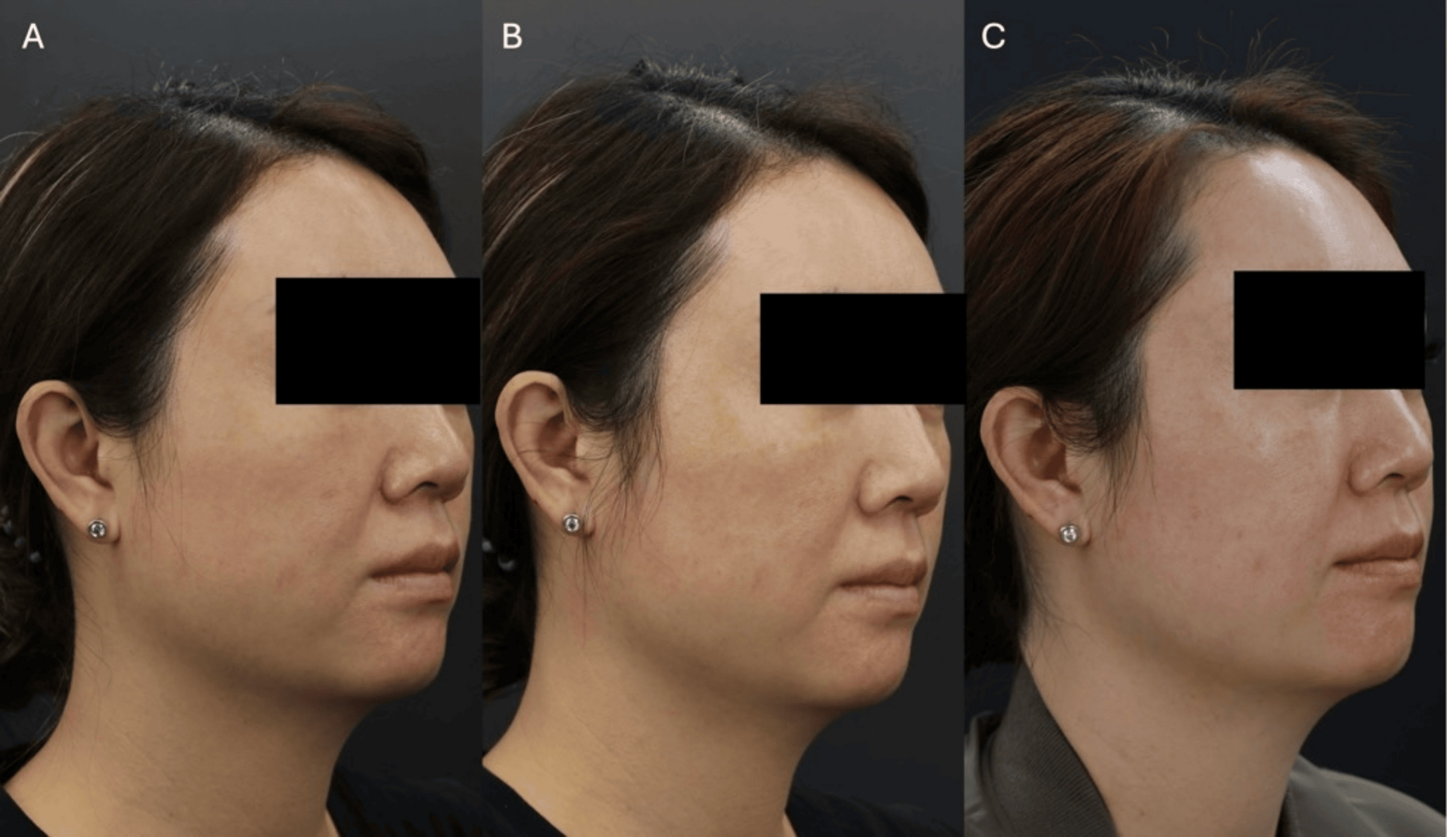
Patient case photographs courtesy of Dr. Yui Lam. A 42-year-old lady received a total of 215 lines of Ultherapy, with a 1.5 mm transducer, followed by 1.5 mL of CaHA-CMC (1:2) evenly spread across the skin, and is shown at baseline (A), one (B), and six months (C) post-treatment for skin quality improvements. Written informed consent to include this image in an open-access article was obtained from the patient. CaHA-CMC: calcium hydroxyapatite-carboxymethylcellulose

MFU-V treatment with a 1.5 mm transducer has been combined with a subdermal wash of CaHA-CMC (1:1 or hyper-diluted 1:2) for a dual-modality dermal regeneration (Figure 6). In CaHA-CMC-treated patients with prior MFU-V exposure, pore and skin texture improvements were visible at one month and six months (Figure 7). Combination treatments can be sequenced either by injecting hyperdiluted CaHA-CMC immediately after MFU-V treatment if both procedures are to be done on the same day or by performing MFU-V at least four weeks after CaHA-CMC injection if treatment sessions are to be spaced apart. A recent histological study [54] demonstrated a greater degree of improvement in superficial musculo-aponeurotic system (SMAS) and dermal layer thickness with same-day MFU and CaHA-CMC treatments than with MFU and CaHA-CMC injections spaced one month apart. This outcome suggests a potential synergistic effect resulting from the application of both modalities within a short interval. MFU and CaHA-CMC both improve dermal collagen levels through different mechanisms of action, and some studies have shown that the combination of both modalities was safe and effective in enhancing overall skin quality [38,39,55-57]. MFU utilizes thermal coagulation points (TCPs) to denature collagen, resulting in a wound-healing cascade [58] involving collagen remodeling [59,60]. The addition of CaHA introduces more mechanical cues through its regenerative scaffolds to induce further collagen and elastin production within the same area. The CaHA particles provide a scaffold for fibroblast attachment and activation and facilitate both neocollagenesis and neoelastogenesis [61].

## Discussion

For almost two decades following its approval for nasolabial fold correction [40,62], APAC physicians have been incorporating CaHA-CMC into their multimodal approaches to treat facial aging and leverage the regenerative benefits of CaHA. In recent years, the ability of CaHA-CMC to stimulate neocollagenesis has been shown to be part of a regenerative process rather than an inflammatory pathway [63].

CaHA-CMC and MFU-V both support dermal remodeling of lax skin, leading to its tightening and lifting [56,64-67]. MFU can heat subcutaneous tissues to optimal temperatures (~60°C) for the induction of neocollagenesis [56,59,60] and elastogenesis [58,60,68] via the body’s natural wound-healing response, resulting in MFU-induced collagen remodeling in mid-to-deep dermal and subdermal reticular layers [60,69,70]. CaHA microspheres make direct contact with fibroblasts [71], resulting in further fibroblast stimulation and neocollagenesis [39,57,69,72-74]. Since MFU-V and CaHA-CMC use different mechanisms to stimulate neocollagenesis [56], combining both modalities in the same area leads to a synergistic enhancement of neocollagenesis [39,55].

When treating the clinical signs of aging, physicians can choose between regenerative biostimulatory agents that tend towards healthy tissue renewal or replacement biostimulants that are focused on neocollagenesis. While replacement via neocollagenesis can replace volume loss and smooth out wrinkles and furrows, it might not optimally restore normal, healthy, pliable tissues. In fact, excessive and misaligned neocollagenesis [75,76] will result in ECM and cell dysfunction [77-79]. This is exemplified in fibrotic scars [80,81] with poor pliability and blood circulation [82]. Injection of biomaterials (e.g., CaHA, hyaluronic acid (HA), PLLA, PCL) immediately changes tissue mechanical tension, stretching the ECM and indirectly upregulating or ‘awakening’ cells [12,83,84]. However, PLLA fillers subsequently follow inflammatory pathways that lead predominantly to collagen production but can also precipitate inflammatory tissue reactions [85,86] and foreign body granulomas [87,88]. In contrast, CaHA microspheres interact directly with fibroblasts, providing biomechanical cues [71] likely through direct mechanotransduction mechanisms and regenerative immunological pathways to drive quiescent cells (fibroblasts) toward regenerative (not replacement) pathways without a chronic granulomatous reaction and the formation of granulomas [4] seen with PLLA [85] and HA fillers [88]. CaHA-CMC also produces collagen that replaces collagen type III (Col III) with the more mature collagen type I (Col I) [89]. Alongside this, CaHA also produces other essential ECM components (e.g., proteoglycans, glycosaminoglycans) [37]. This structural renewal of the ECM is associated with the functional improvements of dermis thickening, increased pliability, and angiogenesis [90,91].

Filler injection initiates an innate immune response that is key for biostimulation and subsequent fibroblast physiological responses. Upon injection, the biomaterial is immediately coated with plasma proteins, including gamma globulins, albumin, fibronectins, and thrombins. A biomaterial’s mechanophysical properties, particularly its smoothness, shape, size, electric charge, nanotopography, chemistry, and plasticity, affect immune cell activation and plasma protein adhesion to the biomaterial. This surface protein deposition drives subsequent responses by macrophages, which take on either pro-inflammatory M1 or pro-reparative/regenerative M2 phenotypes [63]. During inflammatory [92,93] responses to a biomaterial like PLLA, M1 macrophages secrete inflammatory cytokines and attract additional immune cells to initiate a foreign body response. When the biomaterial is too big (>100 uM) for engulfment by macrophages, foreign body giant cells (FBGCs) may form and work together with reparative M2 macrophages to direct fibroblasts to produce collagen to encapsulate the foreign body. This type of repair essentially replaces it with a predominantly single tissue component and can potentially lead to fibrosis and a stiff, non-functioning ECM. It is seen clinically when tissues have become fibrotic, non-pliable, and are difficult to dissect in surgical procedures. In contrast, the authors theorize that CaHA-CMC microspheres are recognized by the mammalian immune system as ‘immune-friendly,’ not as foreign bodies requiring elimination, and that this may be partly attributed to their smoothness, roundness, and chemical similarity to the biological bone matrix [94]. In vitro studies indicate that CaHA-CMC does not initiate pro-inflammatory foreign body pathways but instead follows non-inflammatory, pro-regenerative M2 pathways that activate fibroblasts to increase the production of vital ECM components (e.g., collagen, elastin, proteoglycans). The various immune pathways taken by biostimulators were demonstrated in vitro [63] when M1/M2 macrophages stimulated by Radiesse® (CaHA-CMC; Merz North America, Raleigh, NC, USA) expressed none of the 40 tested pro-inflammatory cytokines, whereas M1 macrophages stimulated by PLLA (Sculptra®; Galderma, Lausanne, Switzerland) expressed five pro-inflammatory cytokines (sTNFR2, CCL1, MIP-1α, MIP-1β, and IL-8), and M2 macrophages expressed three of the pro-inflammatory cytokines (MIP-1α, MIP-1β, and IL-8). PLLA particles also demonstrated flaky, sharp edges and heterogeneous sizes, which may partially explain their high inflammatory potential compared to CaHA-CMC. The smooth surface of CaHA-CMC is an important factor in determining the immunological response to the CaHA [95]. However, poly-D, L-lactic acid (P-DLLA), which is morphologically smooth, can still recognized as non-self by the immune system, leading to a pro-inflammatory response. The particle morphology is only one of the many physicochemical properties that will determine the subsequent immunological response. One characterization study [94] found that CaHA-CMC particles appeared smooth (without surface imperfections), whereas up to 20% of CaHA/HA particles (HarmonyCA; Allergan Aesthetics, CA, USA) were fissured and fragmented. Moreover, primary fibroblasts co-cultured for up to seven days with increasing concentrations of CaHA-CMC or CaHA/HA showed significant increases in Col III levels, but only CaHA-CMC increased Col I [94].

CaHA fillers boost collagen production [96,97]; however, when trying to renew a patient’s ECM function, physicians need to consider the different types of collagen fibers, their ratios, and how they structurally form a 3D matrix with other proteins. Col I comprises 75%-80% of normal skin, while Col III and small amounts of Col IV, V, or VI [8,98,99] comprise the rest. Notably, hypertrophic scars have more Col III than Col I [100], and aging is characterized by a shift in collagen production from Col I to III that results in disarrayed Col I fibrils [101,102] and less elastin. When selecting biostimulators, a haphazardly induced and excessive production of any one type of collagen risks the creation of a stiff, non-functional ECM that will not support its associated cells and should not be a goal of aesthetic treatments.

Aging impacts multiple facial regions and leads to a need for aesthetic treatments for multiple skin quality issues and underlying volume changes. Consequently, physicians must develop an understanding of how the individual face layers and regions interconnect [103], how multiple aesthetic tools and techniques are used for different indications, and how to incorporate these considerations into customized, whole-face, biostimulatory treatments. Such global, multimodal approaches [104] address multiple areas within a single or consecutive treatment session, and since treating one facial area can improve others, this strategy produces more visible and natural facial rejuvenation [104,105], with near-doubled patient well-being and satisfaction [103,106-109].

The regenerative properties of CaHA-CMC [4,38] have been clinically leveraged to treat tired and aged appearances and improve skin quality [40-47] in Asian patients. Asian patients generally have thicker skin [110,111], which is commonly affected by issues such as enlarged pores and hypertrophic and keloid acne scars [112], for which treatments are sought. However, Asian patients are generally more culturally conservative and seek regenerative treatments that preserve discrete facial features while avoiding excessive outcomes like overfilling. The Asian patients treated with CaHA-CMC by our physicians ranged in age from the late thirties to early seventies, consistent with studies showing that Asian patients generally begin aesthetic treatments earlier [113] to address undesirable facial features resulting from underlying facial anatomy and structural deficiencies. With age [114], these Asian patients’ aesthetic concerns change [115]. Those between 25 and 40 years old are usually bothered by problems with skin tone, pigmentation, lower-face contours, and decreased skin elasticity and firmness. Early aging and ethnicity-related concerns, such as non-ideal, masseter-related face shapes and shallow nasal shapes [113,116], also change by 31-40 years to concerns about worsening tear troughs, malar volume deficiencies, and nasolabial folds. By 41-55 years, problems arise with upper face lines and volume loss in tear troughs and malar areas, while jowls, sagging eyelids, and malar volume loss affect those older than 55 years. As facial aging results from the loss of volume and tension [54] in superficial and deep fat compartments [117], combining MFU-V and CaHA improves dermal and subdermal connective tissue architectures, visibly improving multiple aesthetic issues, including uneven contours and loose skin [54]. Treating with MFU-V immediately before regenerative biostimulation with CaHA-CMC was the most effective means of thickening the epidermal, dermal, and SMAS layers [54]. This treatment sequence potentially activated fibroblasts most optimally and maximized neocollagenesis [54] to thicken the SMAS and retinacula cutis. Consequently, subdermal foundations for overlying skin were subjectively found to be more stable, with more cohesive interactions between lateral face layers, reduced soft tissue descent, and strengthened dermis-subdermis attachments. Patients ultimately experience tighter, firmer, less sagging skin and better facial contours. An APAC study on current trends in skin quality treatments also revealed skin surface evenness and enlarged pores as key clinical concerns encountered in Asian patients by healthcare practitioners. Accordingly, a multimodality, multi-layer, “inside-out” approach comprising diluted CaHA-CMC, MFU-V, intradermal botulinum toxin A, and other non-surgical aesthetic procedures was proposed [114]. With growing awareness of facial overfilled syndrome due to accumulative overuse of dermal fillers, patients and practitioners in APAC are now seeking natural-looking results, with a shift towards more regenerative procedures instead of relying solely on HA fillers for volumization and lifting [118].

While CaHA fillers are effective in facial rejuvenation, patient variables such as age, skin type, and baseline skin condition can affect outcomes. Older patients with more pronounced signs of aging, including volume loss and skin laxity, may require more filler or treatment sessions to achieve the desired results. Patients with thinner, more delicate baseline skin thickness may need more care during injection to avoid the development of visible lumps or irregularities [119], while those with thicker skin may be able to accommodate more filler and also develop more visible results. Pre-existing skin conditions such as acne or rosacea may affect responses to CaHA fillers and require additional considerations during treatment planning [120,121]. Age, skin type, and baseline skin condition should thus be thoroughly assessed to allow treatment plans to be customized and patient expectations to be managed.

This report was limited by its meeting nature and would benefit from the conduct of a comprehensive and controlled study across broader Asian populations and with control, including a filler product with a similar mechanism of action. Also, the clinical endpoints that we presented are subjective, and further studies would benefit from more objective measurements, such as cutometry or three-dimensional tissue quantification. In addition, further long-term follow-up of the patients is needed to validate the durability of their treatment effects and outcomes.

## Conclusions

Our experts have used CaHA-CMC both as monotherapy and in combination with other treatment modalities to achieve the multifaceted aesthetic goals of volumization, contouring, and regeneration in patients from across APAC. Together, their clinical experience provides evidence that CaHA-CMC is versatile and effective and grants durable benefits beyond simple cosmetic enhancements. In older patients seeking treatment for skin laxity, wrinkles, crepiness, and volume loss, CaHA-CMC enables physicians to offer a single, minimally invasive product that yields numerous visible aesthetic improvements. Unlike conventional dermal fillers, exploiting the regenerative qualities of CaHA-CMC effectively resolves age-related aesthetic issues in a natural and durable manner, allowing patients to emphasize their own unique features.
